# Supplementary figures and images for: Addition of a carboxy-terminal tail to the normally tailless gonadotropin-releasing hormone receptor impairs fertility in female mice
Source: eLife. 2021 Dec 23;10:e72937. doi: 10.7554/eLife.72937 (PMC8741216; doi:10.7554/eLife.72937)

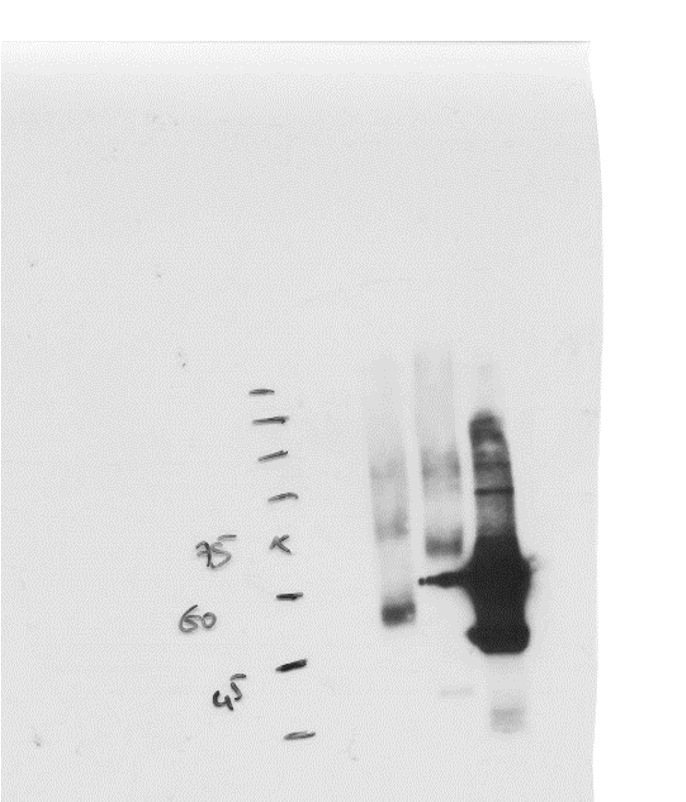

Supplement: Figure 6—figure supplement 1—source data 1. [file elife-72937-fig6-figsupp1-data1.zip › Figure 6-figure supplement 1 panel A-source data 1.png]

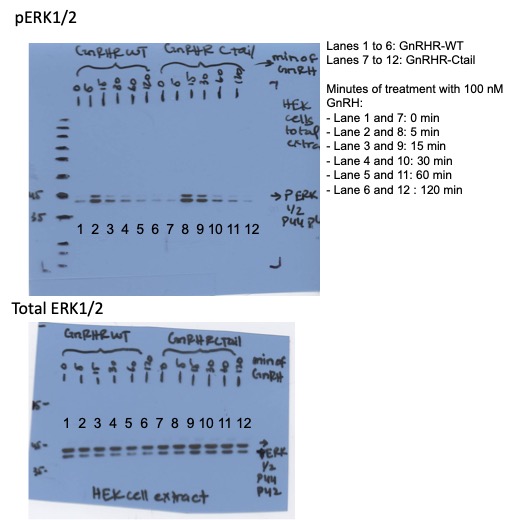

Supplement: Figure 7—source data 1. [file elife-72937-fig7-data1.zip › Figure 7A-source data 1.jpg]

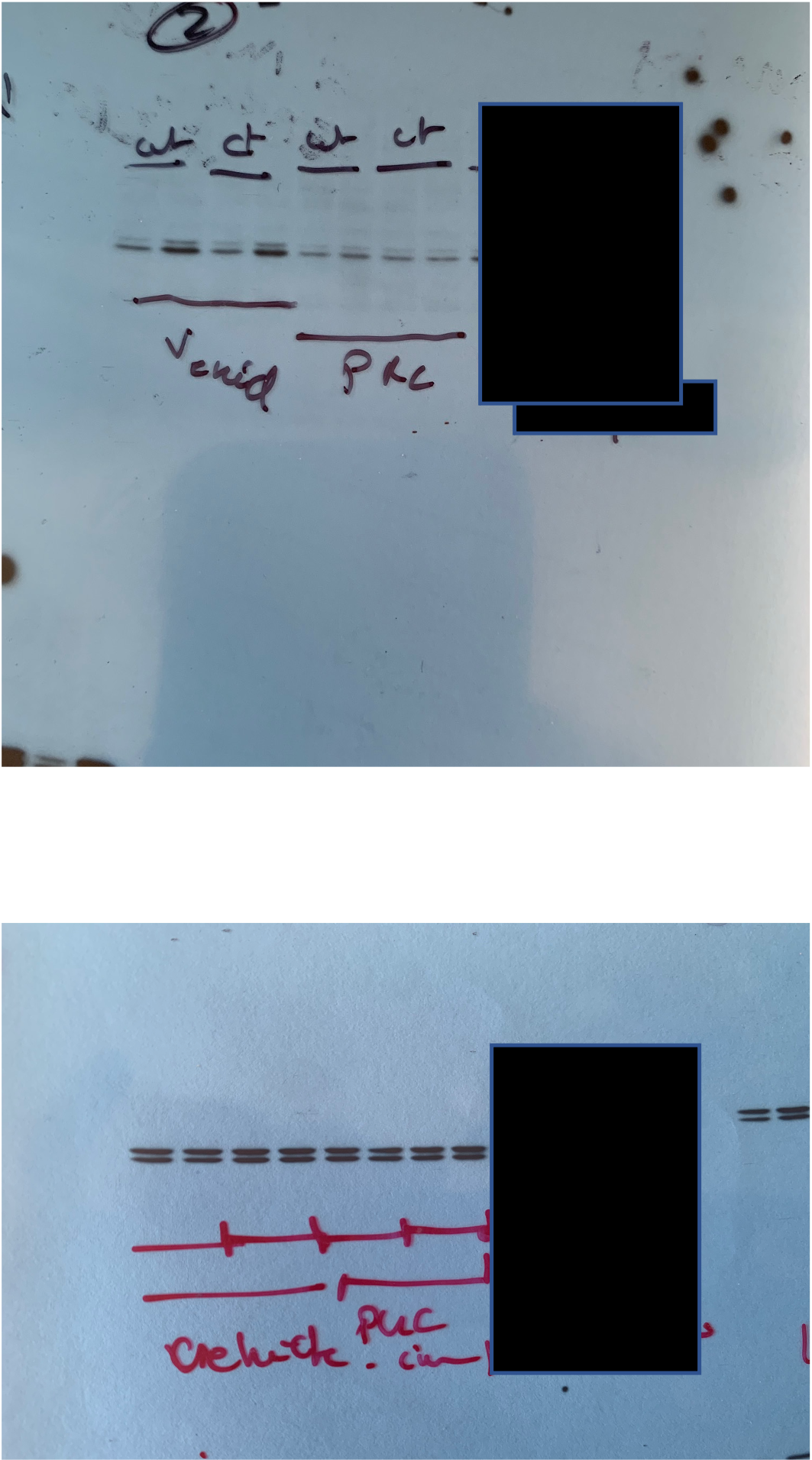

Supplement: Figure 7—source data 2. [file elife-72937-fig7-data2.zip › Figure 7C-source data 1.png]

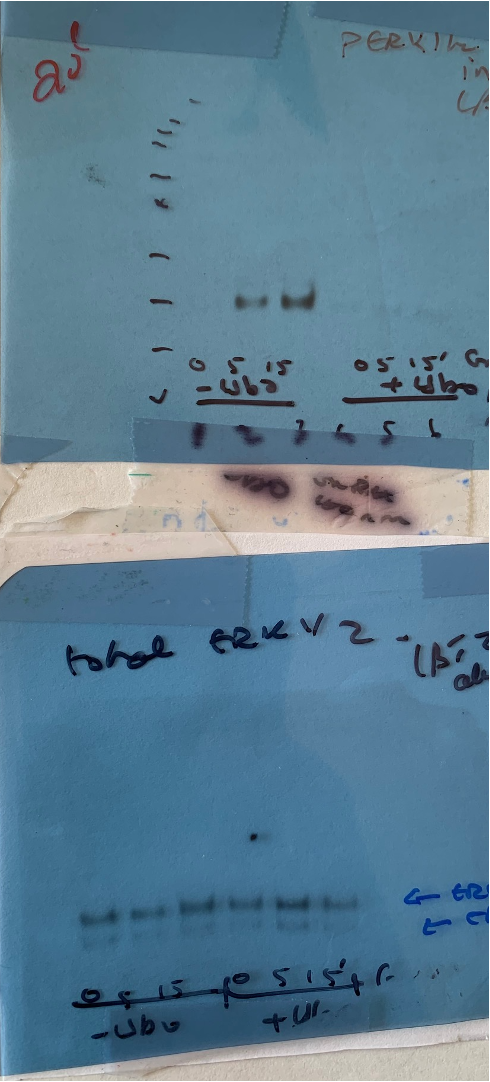

Supplement: Figure 7—figure supplement 1—source data 1. [file elife-72937-fig7-figsupp1-data1.zip › Figure 7-figure supplement 1 panel A-source data 1.png]

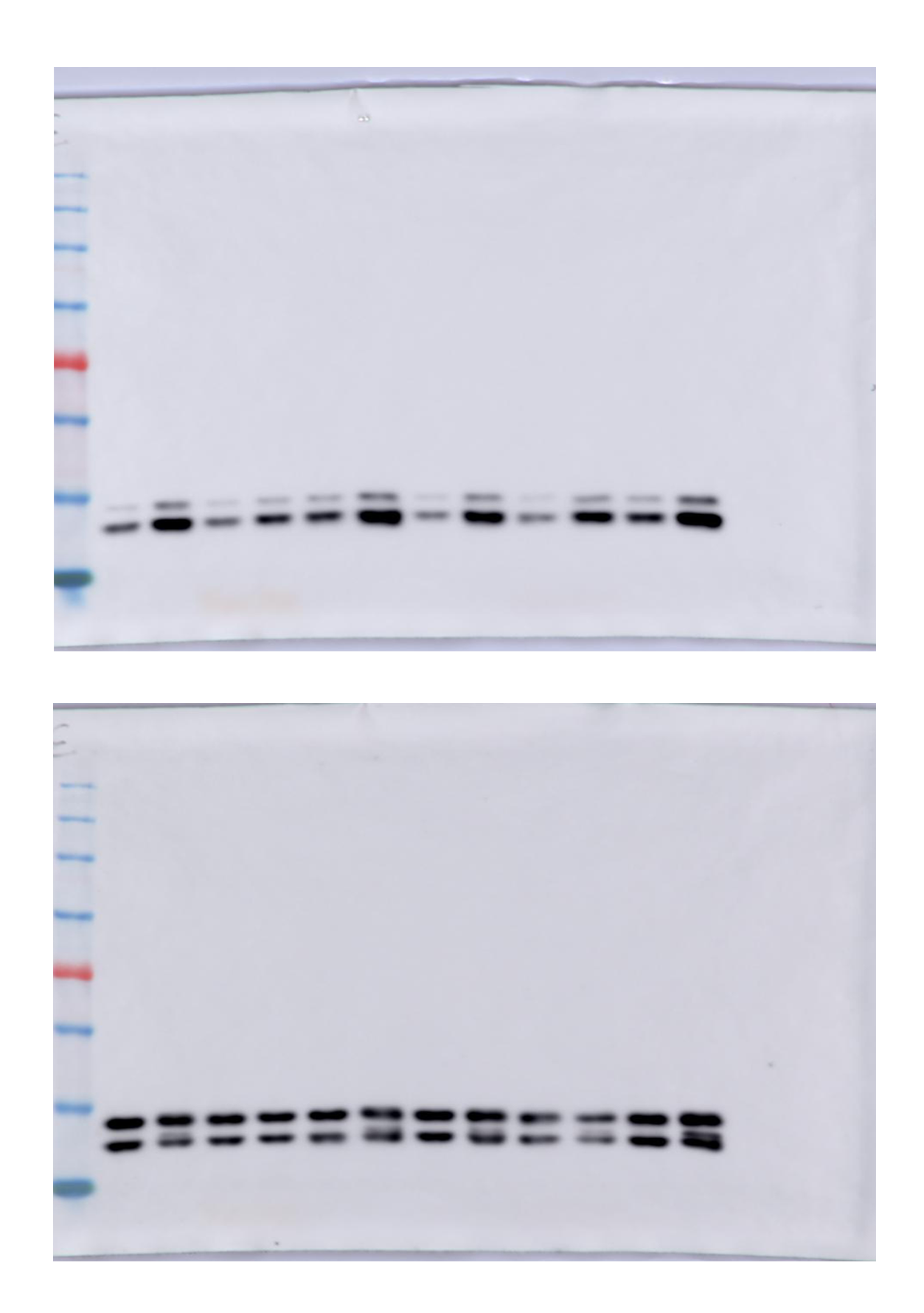

Supplement: Figure 7—figure supplement 1—source data 2. [file elife-72937-fig7-figsupp1-data2.zip › Figure 7-figure supplement 1 panel C-source data 1.tif]

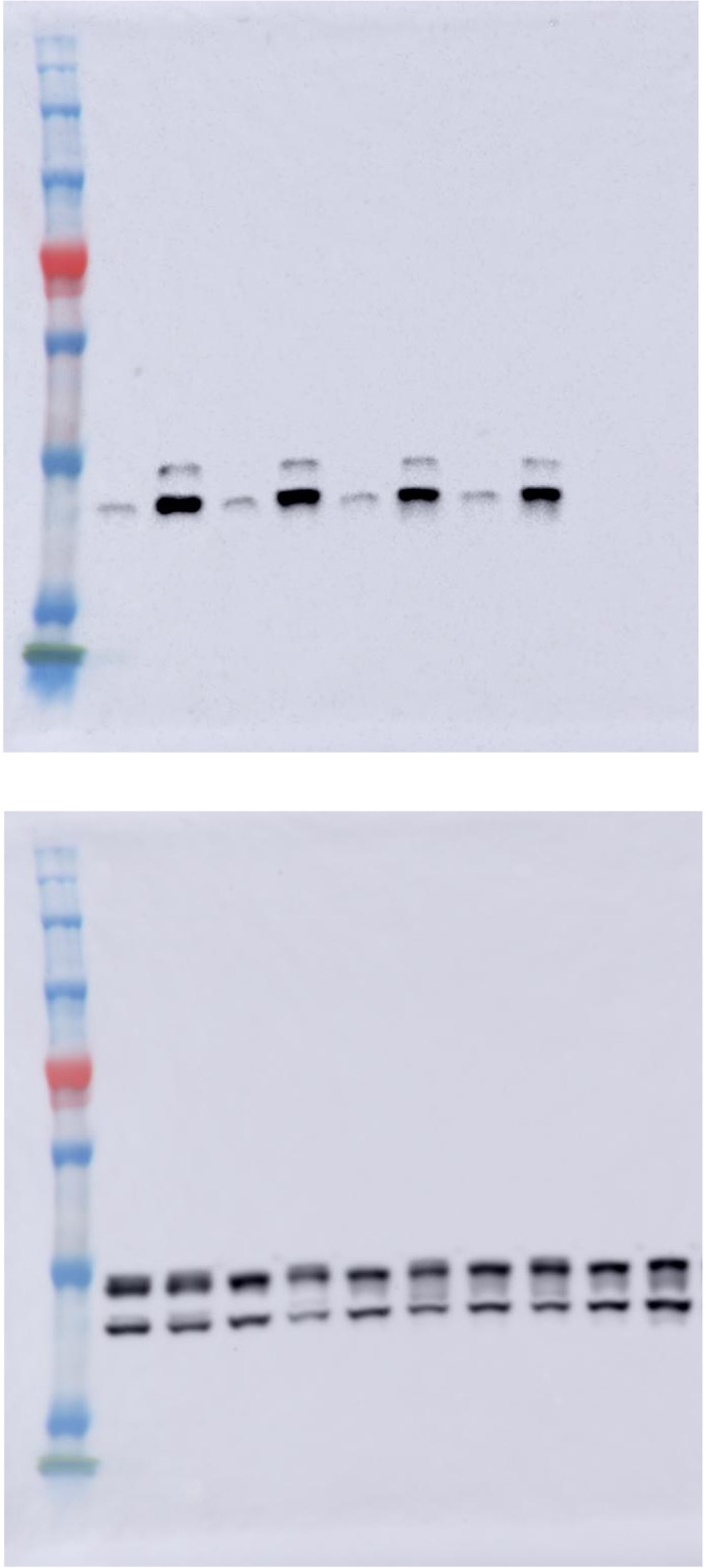

Supplement: Figure 7—figure supplement 1—source data 3. [file elife-72937-fig7-figsupp1-data3.zip › Figure 7-figure supplement 1 panel E-source data 1.jpg]

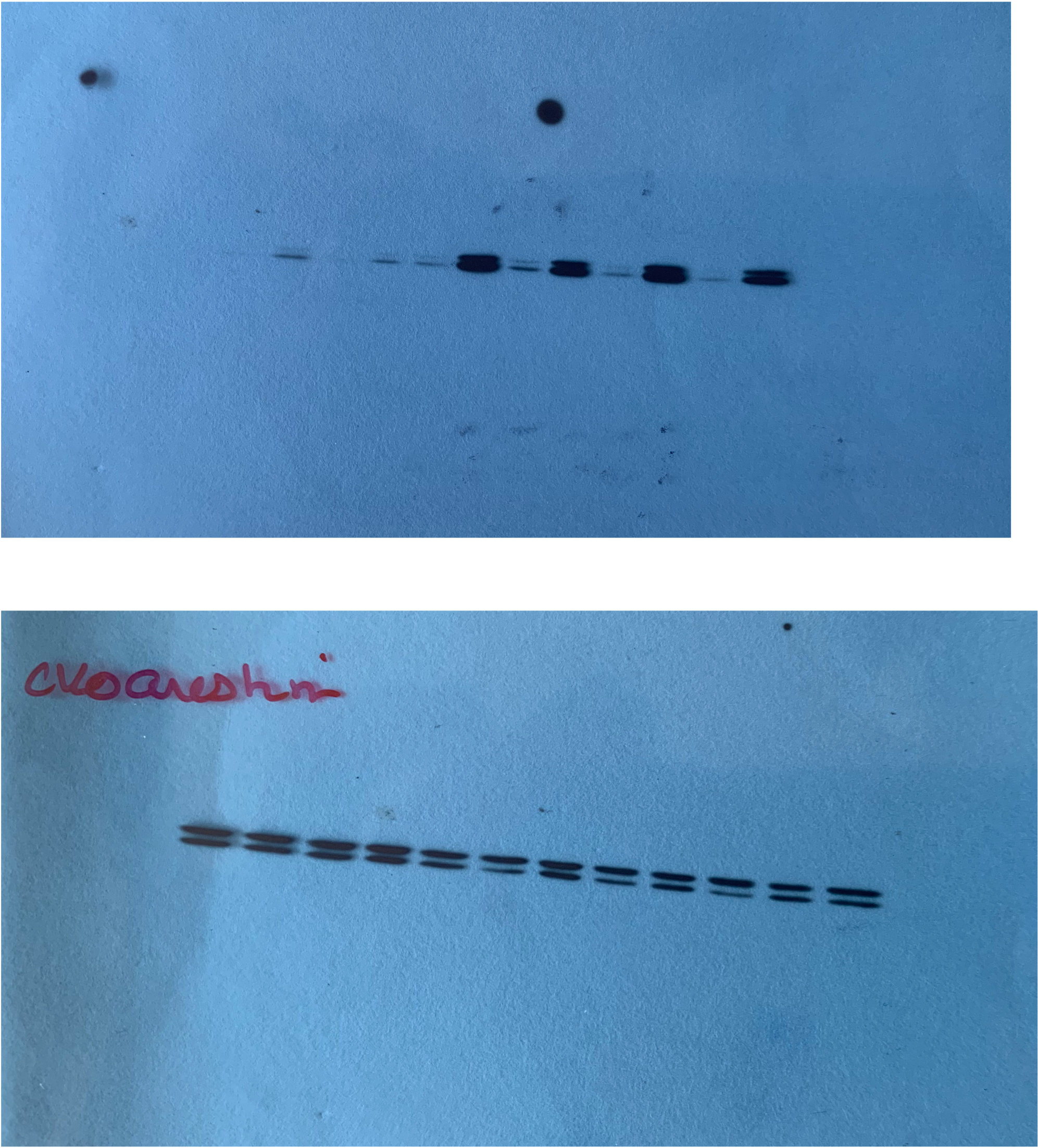

Supplement: Figure 7—figure supplement 2—source data 1. [file elife-72937-fig7-figsupp2-data1.zip › Figure 7-figure supplement 2 panel C-source data 1.png]

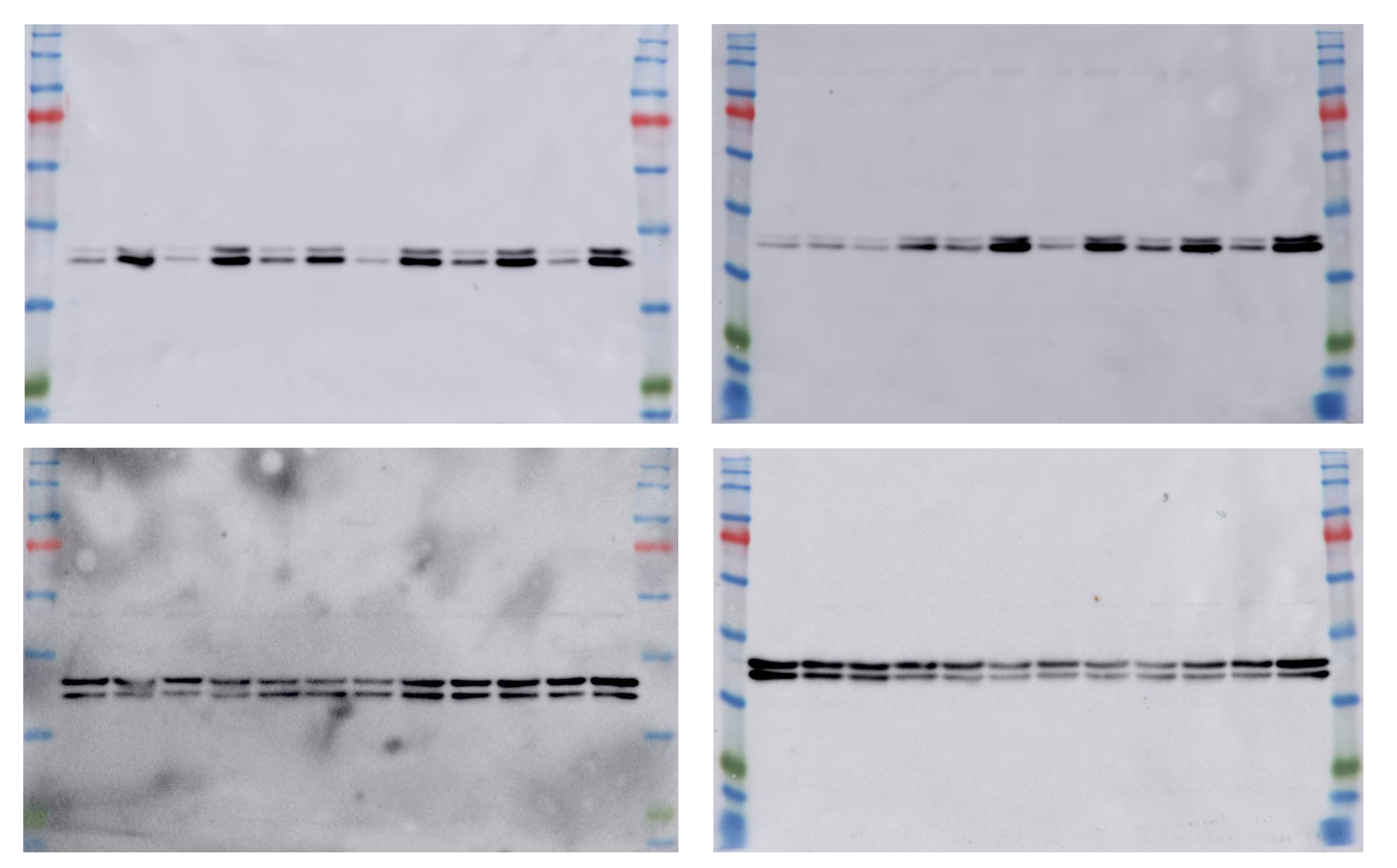

Supplement: Figure 10—source data 1. [file elife-72937-fig10-data1.zip › Figure 10C-source data 1.tif]
